# A biopsychosocial model of severe fear of COVID-19

**DOI:** 10.1371/journal.pone.0264357

**Published:** 2022-02-28

**Authors:** Patrick Nürnberger, Dirk von Lewinski, Hans-Bernd Rothenhäusler, Celine Braun, Patrick Reinbacher, Ewald Kolesnik, Andreas Baranyi

**Affiliations:** 1 Department of Psychiatry and Psychotherapeutic Medicine, Medical University of Graz, Graz, Austria; 2 Department of Internal Medicine Division of Cardiology, Medical University of Graz, Graz, Austria; 3 Department of Orthopaedics and Traumatology, Medical University of Graz, Graz, Austria; Faculty of Health Sciences - Universidade da Beira Interior, PORTUGAL

## Abstract

**Introduction:**

COVID-19 is a respiratory infection that causes not only somatic health issues, but also frequently psychosocial burdens. The aims of this study were to investigate biopsychosocial factors that might further aggravate fear of COVID-19, and to establish a biopsychosocial model of severe fear of COVID-19.

**Methods:**

368 participants were included in this study. Biopsychosocial factors observed comprised biological factors (somatic risk), psychological factors (state/trait anxiety, physical symptoms of anxiety, severe health anxiety, specific phobias, depression), and psychosocial factors (social support, financial losses, social media consumption, social contacts with COVID-19 infected people). Psychometric questionnaires included State-Trait Anxiety Inventory, Beck’s Anxiety Inventory, Whiteley-Index / Illness Attitude Scales, Specific Phobia Questionnaire, WHO-5 and Social Support Survey.

**Results:**

162/368 (44.0%) participants had almost no fear, 170/368 (46.2%) participants had moderate fear, and 45/368 (12.2%) participants had severe fear of COVID-19. Female participants showed higher levels of fear of COVID-19 than male participants (gender: χ2 = 18.47, p<0.001). However, the level of fear of COVID-19 increased in male participants when they had contact with people who were infected with COVID-19, while in contrast the level of fear of COVID-19 decreased in female participants when they had such contacts [ANCOVA: fear of COVID-19 (contact x gender): F(1,363) = 5.596, p = .019]. Moreover, participants without relationships showed higher levels of fear of COVID-19 (marital status: χ2 = 14.582, p = 0.024). Furthermore, financial losses due to the COVID-19 were associated with higher levels of fear of COVID-19 [ANCOVA: fear of COVID-19(financial loss x gender): F(1, 363) = 22.853, p< .001]. Multiple regression analysis revealed female gender, severe health anxiety (WI-IAS) and state /trait anxiety (STAI) as significant predictors of severe fear of COVID-19.

**Conclusion:**

In this study significant predictors of severe fear of COVID-19 were female gender, pre-existing state and trait anxiety, as well as severe health anxiety. The finding of significant predictors of fear of COVID-19 might contribute to detect people who might suffer most from severe, overwhelming fear of COVID-19 at an early stage.

## 1. Introduction

### 1.1. Overview and introduction

Coronaviruses accompany humans, mostly causing mild common cold symptoms. However, some coronaviruses are able to cause severe somatic complications such as pneumonia, respiratory failure and death. Those coronavirus-variants consist of SARS-COV, MERS (Middle Eastern Respiratory Syndrome) and a new variant of 2019 that was named SARS-COV2 and causes the disease COVID-19 (Coronavirus Disease of 2019). It is suspected that the origin of the virus has been in Wuhan, China, where the virus managed to transmit from animals (suspected animal being the pangolin) in a live animal market. From Wuhan, China the virus promptly found its way to spread around the world, causing a worldwide pandemic [1].

The COVID-19 pandemic hit the world to an unpredictable extent. Even though the threat of the appearance of such a pandemic at any time was present, not much precaution and preventive strategies were made. This turned out to be a mistake, as COVID-19 not only causes mild cold-like symptoms, but sometimes also life-threatening severe pneumonia and its possible complications such as multi-organ failure. Thus many people were found to need hospital care or even intensive medical care [1]. Preventive strategies, meaning sanitary concepts in order to organize quarantines and to contain the spread of the virus as well as an accelerated development of therapeutic strategies and vaccinations, were needed. In order to manage the pandemic, to keep medical services at work and to maintain resources for non-COVID-19 emergencies, as well as COVID-19 patients, several health policy steps were needed. As the world saw high numbers of new COVID-19 infections as well as already high numbers of infected people and people dying of/with COVID-19, pronounced health policy measures have been implemented worldwide. This was handled in different ways, mostly with hygienic measures (face masks, repetitive disinfection) and social distancing (no touching, keeping physical distance, reduced social contacts) as well as repeated worldwide lockdowns [with closure of shops and the recommendation to stay at home and to work from home (“homeoffice”) whenever possible]. A worldwide pandemic might cause psychiatric diseases to rise and might decrease the mental health and mental wellbeing of both, healthcare workers as well as the general public. This was already seen in previous outbreaks of pandemic diseases such as SARS-COV in 2003 or Ebola [2–4]. This also applies to the COVID-19 pandemic—in fact, many people in numerous countries were confronted with a lot of uncertainty, a lack of information and even perplexity [5]. Those pandemic fighting measures, most of all the social distancing and isolation of people, but as well other factors such as severe fear of COVID-19, feelings of uncertainty and fear of the future or potential financial problems related to job losses due to COVID-19 caused mental health issues of many people worldwide. Thus, there is a rise in the numbers of mental health diseases such as anxiety and depression. It is possible that these negative mental health effects will be amplified in the long run, as the pandemic lasts since 2020. However, many adverse mental health issues are seen already, and the fact that there might not be enough capacity to care for all those newly developed mental health issues should be alarming for the future [6, 7].

Another severe health-related issue that is related to fear of COVID-19 and the pandemic is the people’s avoidance to seek indicated medical help caused by the fear of an infection with SARS-COV2, e.g. in the doctors’ waiting rooms, the hospital or while being treated (physical contact) by their doctors. This was shown in lower observed incidences of non-COVID19 related diseases such as acute myocardial infarction or pulmonary artery embolism, where numbers were significantly lower than in pre-COVID-19 times. This leads to the hypothesis that people suffering from non-COVID-19 diseases often might have been underdiagnosed and undertreated since the outbreak of the pandemic [8, 9].

Moreover, not only the environmental factors of the COVID-19 pandemic may cause a rise in anxiety, depression and posttraumatic stress symptoms, but also the potential somatic consequences of COVID-19 and the Long-COVID syndrome [10, 11].

Social support might be a protective factor that might decrease the amount of COVID-19-induced anxiety and depression [12].

Given the fact that many people worldwide were faced with lockdowns and social distancing, the use of social media often offered an important source for information updates concerning COVID-19. However, social media may also act as an amplifier of fear of COVID-19. Social media can be seen as beneficial, as it helps to spread information faster and to a broader audience [13]. However, spread of misleading information, fake news and chevy in those respective social media platforms has been observed. This could possibly affect vulnerable people who use social media and their levels of fear of COVID-19 might increase, especially as they consume even more social media in times where they cannot meet their friends and family and want to stay social and connected, at least in a digital way [14].

### 1.2. Aims of the study

A biopsychosocial approach might be beneficial in the explanation of severe fear of COVID-19 and might help to identify those people who are affected by overwhelming fear of COVID-19 at an early stage. The first aim of this study was to investigate potential biopsychosocial factors that might further aggravate fear of COVID-19, including a.) *biological factors* (individual pre-existing somatic risk factors), b.) *psychological factors* [pre-existing anxiety (state/trait anxiety, physical symptoms of anxiety, severe health anxiety); specific phobias; mental wellbeing/signs of depression] and c.) *psychosocial factors* (general social support, financial losses due to the COVID-19 pandemic, social contact with people who were infected with COVID-19 and use of social media).

Further aims of this study were to identify significant biopsychosocial predictors that might further aggravate severe fear of COVID-19 and to establish a biopsychosocial model of fear of COVID-19.

**Fig 1:**
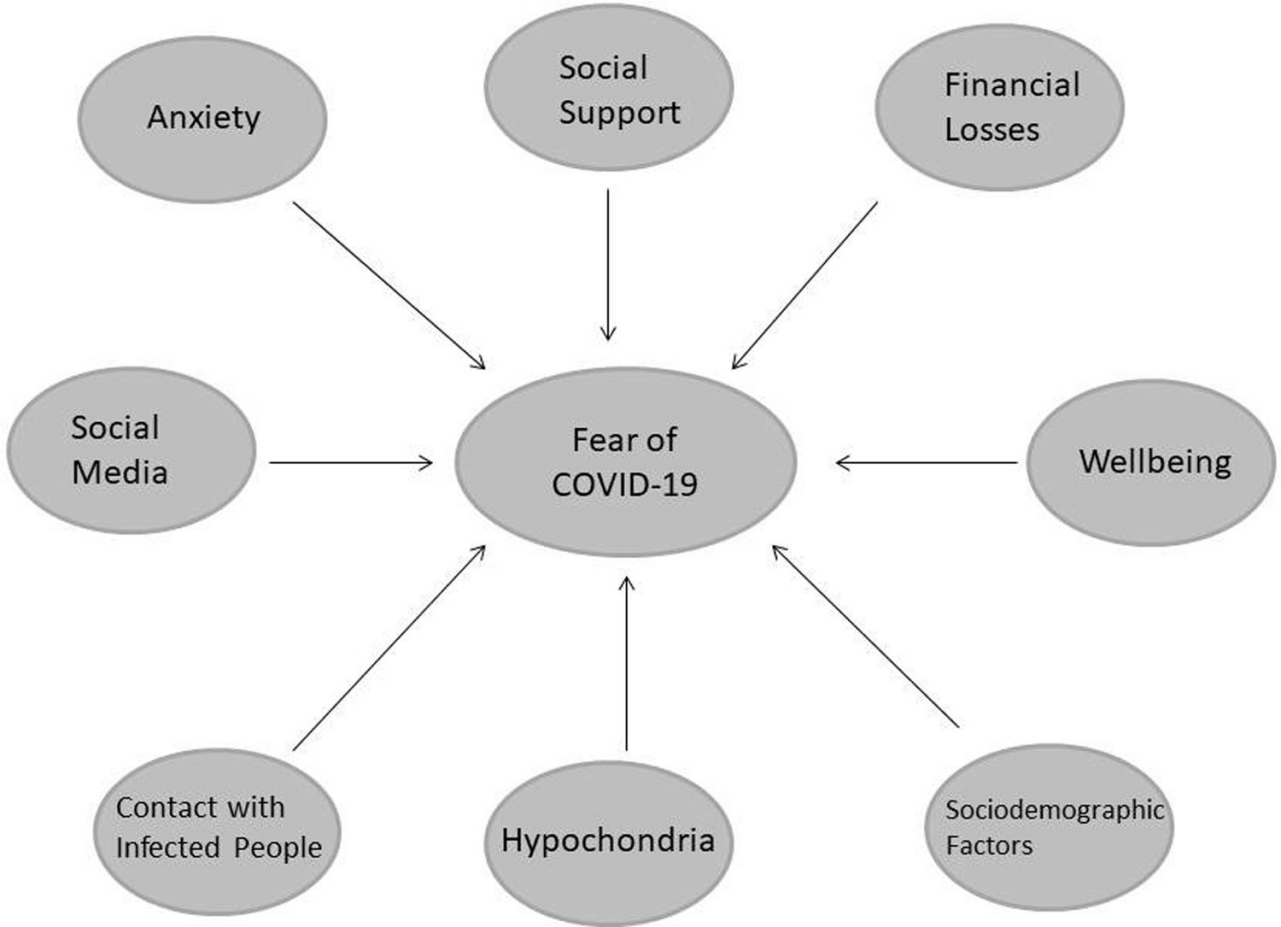
Potential biopsychosocial factors assessed in this study that might impact the level of fear of COVID-19.

Fig 1 shows potential biopsychosocial factors of severe fear of COVID-19 observed in this study (Fig 1).

## 2. Methods

### 2.1. Procedures

368 participants were included in this study (the overall response rate was 95%). Participants included Austrian and German citizens and the study period was from April 18^th^ 2020 to April 5^th^ 2021. All participants answered author compiled single items for sociodemographic data, use of social media, media consumption, financial losses/job losses due to COVID-19 and somatic risk factors for severe COVID-19 course of disease. The research battery consisted of well-validated psychometric questionnaires to objectify a.) the amount of fear of COVID-19 (Severity Measure for Specific Phobia adapted for COVID-19 [15]), b.) pre-existing anxiety [physical symptoms of anxiety (Beck’s Anxiety Inventory, BAI [16]), state and trait anxiety (State-Trait Anxiety Inventory, STAI [17]), severe health anxiety (Whiteley-Index and Illness Attitude Scales, WI-IAS [18]), specific phobias (Specific Phobia Questionnaire, SPQ [19])], c.) mental wellbeing and signs of depression (WHO-5-Well-being Index [20]) and d.) general social support (Social Support Survey, SSS [21]). All questionnaires were well-validated German versions and the data collection was performed digitally with the online survey system of Red Cap (https://www.project-redcap.org) by the Vanderbilt University, licenced by the Medical University of Graz, 8036 Graz, Austria.

Inclusion criteria: people of all genders, aged 19–90 and informed consent. Exclusion criteria were: age below 19 or over 90, previous positive test for SARS-COV2 (PCR-test). pre-existing psychiatric disorders, diagnosed cognitive impairment (e.g. dementia). The ethical approval and trail registry has been obtained prospectively. The participants did not receive any financial compensation.

### 2.2. Measures

#### 2.2.1. Sociodemographic data

In single items for sociodemographic data participants were asked to provide information about their gender, age, education, marital status, household information and job details.

#### 2.2.2. Biological factors—somatic risk factors for a severe COVID-19 course of disease

In single items considering somatic risk factors for a severe course of COVID-19, participants were asked for old age, obesity, asthma, cardiovascular diseases, autoimmune diseases, liver disease, cancer, pre-existing medication that might impact the immune system.

#### 2.2.3. Mental health factors

*a*.*) Amount of fear of COVID-19*. Severity Measure for Specific Phobia [15] adapted for COVID-19: It measures the level of fear of COVID-19. According to the Severity Measure of Specific Phobia the participants were divided into three groups–almost no fear of COVID-19, some fear of COVID-19 but without any phobic characteristics and strong fear of COVID-19 with potential phobic characteristics. For the COVID-19 adapted version a Cronbach’s alpha of .813 was calculated.

*b*.*) Anxiety*. State/trait anxiety. State-Trait Anxiety Inventory (STAI) [17]: A 4-point Likert scale self-report that consists of 40 questions. It measures two different types of anxiety–state anxiety and trait anxiety in the sense of a personal trait. It sums up to a total score of a minimum of 20 up to a maximum of 80 points, whereas higher scores show greater levels of state/trait anxiety.

Severe health anxiety. Whiteley-Index (WI) [18] and Illness Attitude Scales (IAS) [18]: Both look for severe health anxiety up to hypochondria with results reaching from no severe healthy anxiety to suspected severe healthy anxiety and manifest severe healthy anxiety. The WI uses a dichotomous scoring of items whereas the IAS uses a 0–4 Likert scale.

Physical symptoms of anxiety. Beck’s Anxiety Inventory (BAI) [16]: A self-reporting inventory that is used for measuring the severity of physical symptoms of anxiety. It sums up to a total score of 63 and allows to divide the participants into four groups–no to minimal physical symptoms of anxiety, mild physical symptoms of anxiety, moderate physical symptoms of anxiety and severe physical symptoms of anxiety.

*c*.*) Specific phobias*. Specific Phobia Questionnaire (SPQ) [19]: A 5-point Likert Scale that evaluates different kinds of specific phobias and phobia-like fears.

*d*.*) Wellbeing/signs of depression*. WHO-5 Well-being Index (WHO-5) [20]: A well-established and validated index to evaluate mental wellbeing and signs of depression. Higher scores reflect higher levels of mental wellbeing, lower levels indicate signs of depression.

#### 2.2.4. Psychosocial factors

*a*.*) Social Support*. Social Support Survey (SSS) [21]: The SSS is a measure to evaluate social support, higher scores reflect higher social support.

*b*.*) Social contact with people who were infected with COVID-19*. Participants were asked whether they had previous social contact with people who were infected with COVID-19.

*c*.*) Social media*. Concerning social media use the participants were asked in single items how many social media platforms (Facebook, Instagram, WhatsApp, YouTube, Snapchat, Pinterest, TikTok) they used during the last month. Based on the median participants were divided into two groups: less than 4 different social media platforms and use of 4 or more different social media platforms.

*d*.*) Financial losses due to COVID-19*. Participants were asked if they had financial losses due to due to the COVID-19 pandemic.

## 3. Statistical analyses

Descriptive Statistics are presented as means and standard deviations. All non-parametric data are presented in absolute and relative frequencies. To analyse categorial variables χ^2^-Tests were performed. Continuous data were checked according their normal distribution. The level of fear of COVID-19 was measured with the Severity Measure of Specific Phobia [15], adapted for COVID-19. In order to test if there are statistical significant differences between participants with almost no fear of COVID-19, some fear of COVID-19 and strong fear of COVID-19 regarding pre-existing anxiety [state/trait anxiety (State-Trait Anxiety Inventory [17]); physical symptoms of anxiety (Beck’s Anxiety Inventory [16]); severe health anxiety (Whiteley-Index and Illness Attitude Scales [18])], specific phobias (Specific Phobia Questionnaire [19]), mental wellbeing/signs of depression (WHO-5 Well-being Index [20]) and social support (Social Support [21]) two-way analyses of co-variance (ANCOVAs, group x gender) with preceding Levene tests or box tests to check for homogeneity of variances were performed. Age was used as covariate. In addition, post-hoc tests were performed. To further explore the impact of the covariate age Pearson’s correlation analyses were performed. To determine significant predictors of severe fear of COVID-19 and to establish a biopsychosocial model of severe fear of COVID-19 a linear multiple regression analysis was performed. The depended variable (DV) was the level of fear of COVID-19 (DV: interval scaled Severity Measure of Specific Phobia score [15]) and the potential predictors were: pre-existing anxiety [state and trait anxiety (State-Trait Anxiety Inventory [17]), physical symptoms of anxiety (Beck’s Anxiety Inventory [16]), severe healthy anxiety (Whiteley-Index and Illness Attitude Scales [18])], specific phobias (Specific Phobia Questionnaire^18^),mental wellbeing/signs of depression (WHO-5 Well-being Index [20]), general social support (Social Support Survey^23^), use of social media, financial losses due to the COVID-19 pandemic, history of direct social contact with people infected with COVID-19.

## 4. Results

### 4.1. Sociodemographic characteristics

A total of 368 participants were included. 93/368 (25.3%) participants were male, 275/368 (74.7%) participants were female. The mean age was 33.51 years (SD ±12.1).

### 4.2. Fear of COVID-19

Based on the Severity Measure for Specific Phobia [15] adapted for COVID-19 the participants were split into three groups with different levels of fear of COVID-19: a) people with almost no fear of COVID-19 (n = 162/368, 44.02%), b) people with moderate fear of COVID-19 (n = 161/368, 43.75%), c) people who had severe levels of fear of COVID-19 with potential phobic characteristics (n = 45/368, 12.23%). In the group with almost no fear of COVID-19 there were 54/162 (33,3%) male and 108/162 (66,6%) female participants which means that 54/93 (58.1%) of all male participants showed almost no fear and 108/275 (39.3%) of all female participants showed almost no fear, in the group with moderate fear of COVID-19 there were 38/161 (23,6%) male and 123/161 (76,4%) female participants which means that 38/93 (40.9%) of all male participants and 123/275 (44.7%) of all female participants showed moderate fear of COVID-19 and, and in the group with severe fear of COVID-19 with potential phobic characteristics there were 1/45 (2,2%) male participant and 44/45 (97,8%) female participants which means that 1/93 (1.1%) of all male participants and 44/275 (16%) showed severe fear of COVID-19 with potential phobic characteristic. Female participants showed more severe fear of COVID-19 than male participants (χ2 = 18.47, df = 2, p<0.001).

#### 4.2.1. Fear of COVID-19 and sociodemographic characteristics

*Education*. The level of education did not correlate with the levels of fear of COVID-19 (χ ^2^ = 8.838, df = 8, p = 0.356) (see Table 1).

**Table 1:** Level of education and its impact on fear of COVID-19.

Fear of COVID-19		Compulsory School	Middle School	Apprentice-ship	Secondary School	University	Total
**No or Almost No Fear**	n	9	1	28	46	78	162
	%	52.9%	11.1%	46.7%	39.7%	47%	44.%
**Moderate Fear**	n	6	6	22	58	69	161
	%	35.2%	66.7%	36.7%	50%	41.6%	43.%
**Severe Fear**	n	2	2	10	12	19	45
	%	11.7%	22.2%	16.7%	10.3%	11.4%	12.%
	Total %	100%	100%	100%	100%	100%	100%

*Marital status*. 226/368 (61.4%) of the participants were in a relationship or married, 130/368 (35%) were single, 3/368 (0.8%) were widowed and 9/368 (2.4%) were divorced/separated (see Table 2). People who are in a relationship show significant lower levels of fear than those who are single, widowed or divorced/separated (χ ^2^ = 14.582, df = 6, p = 0.024) (see Table 2).

**Table 2:** Marital status and its impact on fear of COVID-19.

Fear of COVID-19		In a relationship/married	Single	Widowed	Divorced or Separated	Total
**No or Almost No Fear**	n	111	48	0	3	162
	%	49.1%	36.9%	0.0%	33.3%	44.0%
**Moderate Fear**	n	96	58	3	4	161
	%	42.5%	44.6%	100%	44.4%	43.8%
**Severe Fear**	n	19	24	0	2	45
	%	8.4%	18.5%	0.0%	22.2%	12.2%
	Total %	100%	100%	100%	100%	100%

*Household conditions*. There was no significant difference between household conditions and levels of fear of COVID-19 (χ ^2^ = 2.283, df = 4, p = 0.684) (see Table 3).

**Table 3:** Household conditions and its impact on fear of COVID-19.

Fear of COVID-19		Living Alone/ Single Household	Living with Partner	Household With Children	Total
**No or Almost No Fear**	n	46	67	49	162
	%	43%	44.1%	45%	44.0%
**Moderate Fear**	n	44	70	47	161
	%	41.1%	46%	43.1%	43.8%
**Severe Fear**	n	17	15	13	45
	%	15.9%	9.9%	11.9%	12.2%
	Total %	100%	100%	100%	100%

#### 4.2.1. Summary of the results of the psychometric questionnaires depending on the level of fear of COVID-19

**Table 4** summarizes the results (means and standard deviations) of the psychometric questionnaires of male and female participants with almost no fear, moderate fear and severe fear of COVID-19. The results of the psychometric questionnaires are presented in detail in the following chapters.

**Table 4:** Means (±SD) of the psychometric questionnaires of male and female participants with almost no fear, moderate fear and severe fear of COVID-19.

Fear of COVID-19												
ALMOST NO FEAR					MODERATE FEAR				SEVERE FEAR			
	Male (n = 54)		Female (n = 108)		Male (n = 38)		Female (n = 123)		Male (n = 1)		Female (n = 44)	
	Mean	SD(±)	Mean	SD(±)	Mean	SD(±)	Mean	SD(±)	Mean	SD(±)	Mean	SD(±)
**Anxiety**												
BAI	3.13	±4.13	5.97	±7.75	7.58	±9.74	8.76	±7.51	6.00	-	17.59	±13.42
IAS	15.20	±7.20	17.95	±9.71	16.87	±9.10	24.26	±12.48	27.00	-.	33.91	±18.04
WI	1.65	±1.31	1.75	±1.97	2.13	±2.40	3.07	±2.71	5.00	-.	4.80	±3.68
STAI STATE	31.87	±8.85	34.36	±8.19	35.47	±10.45	38.52	±9.16	32.00	-.	52.39	±13.85
STAI TRAIT	31.19	±7.14	37.03	±9.96	34.79	±10.35	39.45	±9.86	33.00	-.	51.41	±13.53
**Specific Phobia**												
SPQ	.20	±.17	.37	±.31	.34	±.26	.48	±.33	.02	-.	.83	±.72
**Signs of Depression**												
WHO-5	32.81	±16.51	37.19	±18.64	37.05	±19.39	44.16	±17.06	44.00	-.	62.45	±23.60
**Social Support**												
SOCIAL SUPPORT	81.92	±15.22	80.47	±16.38	77.98	±15.79	80.01	±15.51	93.42	-.	68.45	±24.42

BAI: Beck’s Anxiety Inventory.

IAS: Illness Attitude Scales.

Social Support: Social Support Survey.

SPQ: Specific Phobia Questionnaire.

STAI State: State-Trait Anxiety Inventory.

STAI Trait: State-Trait Anxiety Inventory.

WHO: WHO-5 questionnaire of wellbeing.

WI: Whiteley-Index.

### 4.3. Biopsychosocial risk factors for severe fear of COVID-19

#### 4.3.1. Biological risk factors and fear of COVID-19

Participants were asked whether they had any of those known somatic risk factors for a severe course of disease of COVID-19: e.g. old age, obesity, asthma, cardiovascular diseases, autoimmune diseases, liver disease, cancer or medication that impacts the immune system. There were 228/368 participants (62%) claiming they did not have any somatic risk factors, 97/368 participants (26.2%) claiming that they had 1 risk factor and 43/368 participants (11.7%) claiming that they had more than 2 somatic risk factors.

Somatic risk factors did not have any impact on the levels of fear of COVID-19 [ANCOVA: fear of COVID-19 (somatic risk factors): F(2, 361) = 2.658, *p* = .071; fear of COVID-19 (gender): F (1, 361) = 19.295, *p*< .001; fear of COVID-19 (somatic risk factors x gender): F (2,361) = 1.446, *p* = .237. The covariate age showed no significant impact (F(1, 361) = 0.652 *p* = .420).]

#### 4.3.2. Psychological factors—Mental health factors and fear of COVID-19

*4*.*3*.*2*.*1*. *Pre-existing anxiety and severe fear of COVID-19*. a.) State anxiety. The STAI-State [17] was used to assess state anxiety. Regarding state anxiety there is a gender independent significant difference between participants with different levels of fear of COVID-19. The higher the levels of fear of COVID-19 were, the higher the scores for the STAI State were. So participants with higher levels of fear of COVID-19 showed higher levels of state anxiety. [ANCOVA: STAI State (fear of COVID-19): F(2, 355) = 5.55, *p* = .004; STAI-State (gender): F(1, 355) = 5.89, *p* = .016; STAI State (fear of COVID-19 x gender): F(2, 355) = 1.64, *p* = .195. The covariate age showed a significant impact (F(1, 355) = 4.48, *p* = .035).]. Correlation analysis showed that the higher the age of a person was, the lower their level of state anxiety was (Pearson’s *r* = -.142, *p* = 0.007).

b.) Trait anxiety. The STAI-Trait (18) evaluates anxiety as a personal trait (trait anxiety). Regarding trait anxiety there is a gender independent significant difference between participants with different levels of fear of COVID-19. The higher the levels of fear of COVID-19 were, the higher the scores for the STAI-Trait were. So participants with higher levels of fear of COVID-19 showed higher levels of trait anxiety. [ANCOVA: STAI-Trait (fear of COVID-19): F(2, 355) = 3.14, p = .045; STAI-Trait (gender): F(1, 355) = 6.68, p = .010; STAI-Trait (fear of COVID-19 x gender): F(2, 355) = 0.870, p = .420. The covariate age showed a significant impact (F(1, 355) = 5.902, p = .016).]. Correlation analysis showed that the higher the age of a person was, the lower their levels of trait anxiety was (Pearson’s *r* = -.170, *p* = 0.001).

c.) Physical symptoms of anxiety during the COVID-19 pandemic. The BAI [16] was used to assess physical symptoms of anxiety. Regarding physical symptoms of anxiety there is a gender independent significant difference between participants with different levels of fear of COVID-19. The higher the levels of fear of COVID-19 were, the higher they scored in the BAI. [ANCOVA: BAI (fear of COVID-19): F(2, 361) = 5.87, *p* = .003; BAI (gender): F(1, 361) = 2.56, *p* = .110; BAI (fear of COVID-19 x gender): F(2, 361) = 0.85, *p* = .43. The covariate age showed a significant impact (F(1, 361) = 5,87, *p* = .003).]. Correlation analysis showed that the higher the age of a person was, the lower their levels of physical symptoms of anxiety were (Pearson’s *r* = -.193, *p*< 0.001).

d.) Severe health anxiety. The WI-IAS [18] was used to evaluate pre-existing severe health anxiety up to hypochondriac tendencies in participants with and without severe fear of COVID-19.

Concerning severe health anxiety there is a gender independent significant difference between participants with different levels of fear of COVID-19. So participants with higher levels of fear of COVID-19 show more symptoms of severe health anxiety. [ANCOVA: Whitely-Index (fear of COVID-19): F(2, 355) = 6.356, *p* = .002; Whitely-Index (gender): F(1, 355) = 0.059, *p* = .809; Whitely-Index (fear of COVID-19 x gender): F(2, 355) = 1.012, *p* = .365. The covariate age showed no significant impact (F(1, 355) = 1.332, *p* = .249).]

There is also a significant difference between participants with almost no fear of COVID-19 and fear of COVID-19 concerning the IAS questionnaire. The more severe the fear of COVID-19 was, the higher the scores in the IAS. [ANCOVA: IAS (fear of COVID-19): F(2, 361) = 5.58, *p* = .004; IAS (gender): F (1, 361) = 1.81, *p* = .180; IAS (fear of COVID-19 x gender): F (2, 361) = 1.35, *p* = .261. The covariate age showed no significant impact (F(1, 361) = 0.796, *p* = .373).]

e.) Specific phobias during the COVID-19 pandemic. Concerning specific phobias in general during the COVID-19 pandemic there is a gender independent significant difference between participants with different levels of fear of COVID-19. Participants with higher levels of fear of COVID-19 also showed higher tendency for specific phobias during the COVID-19 pandemic. [ANCOVA: Specific Phobia Questionnaire (sum of all subcategories of phobia) (fear of COVID-19): F(2, 360) = 4.063, *p* = .018; SPQ (gender): F (1, 360) = 8.696, *p* = .003; SPQ (fear of COVID-19 x gender): F (2, 360) = 1.597, *p* = .204. The covariate age showed no significant impact: (F(1, 360) = 0.964, *p* = .405).]

Concerning phobias for medical procedures (e.g. being stung by a needle) there is no significant difference between people with low fear of COVID-19 and high fear of COVID-19. [ANCOVA: SPQ Medical Phobia (fear of COVID-19): F (2, 360) = 0.946, p = .389; SPQ Medical Phobia (gender): F(1, 360) = 5.998, p = .015; SPQ Medical Phobia (fear of COVID-19 x gender): F (2, 360) = 1.457, p = .234. The covariate age showed a significant impact (F(1, 360) = 5.694, p = .018).]. Correlation analysis showed no significant correlation of age of a person and the levels of medical phobias (Pearson’s *r* = -.082, *p*< 0.116).

*4*.*3*.*2*.*2*. *Mental wellbeing/signs of depression during the COVID-19 pandemic*. The WHO-5 Well-being Index (WHO-5) [20] questionnaire was assessed to evaluate wellbeing/signs of depression in participants with and without fear of COVID-19.

Concerning mental wellbeing/signs of depression there is a gender independent significant difference between participants with different levels of fear of COVID-19. The higher the COVID-19 -fear was, the lower the WHO-5 mental wellbeing scores were and thus people with higher levels of fear of COVID-19 are more likely to also show signs of depression. [ANCOVA: WHO-5 (fear of COVID-19): F(2, 361) = 3.58, *p* = .029; WHO-5 (gender): F(1, 361) = 1.75, *p*< .187; WHO-5 (fear of COVID-19 x gender): F(2, 361) = 0.49, *p* = .611. The covariate age showed a significant impact (F(1, 361) = 13.01, *p*< .001).]. Correlation analysis showed that the higher the age of a person was, the higher their scores in the WHO-5 were (*r* = -.158, *p*< 0.002).

#### 4.3.3. Psychosocial factors and fear of COVID-19

*a*.*) General social support and fear of COVID-19*. The Social Support Survey (SSS) [21] was applied to find out whether participants with different levels of fear of COVID-19 have differences regarding their general social support.

Concerning the Social Support Survey (SSS) there is no significant difference in their general social support between people with different levels of fear of COVID-19. [ANCOVA: Social Support Survey (fear of COVID-19): F(2, 359) = 0.59, *p* = .553; Social Support Survey (gender): F(1, 359) = 1.98, *p* = .161; Social Support Survey (fear of COVID-19 x gender): F(2, 359) = 1.39, *p* = .249. The covariate age showed no significant impact (F(1, 359) = 0.28, *p* = .596).]

*b*.*) Social contact with people infected with COVID-19 and fear of COVID-19*. Participants were asked whether they had previous social contact with people who were infected with COVID-19. There were 208/368 people (56.5%) claiming they did not have any known contacts, whereas 160/368 people (43,5%) claimed that they know people in their surroundings that were infected with COVID-19.

The results of the ANCOVA show that the levels of fear of COVID-19 were dropping in female participants if they had social contact with people who were infected with COVID-19. The results for male participants showed opposite effects–the more contact male participants had with people who were infected with COVID-19 the higher their levels of fear of COVID-19 were.

[ANCOVA: fear of COVID-19(contact): F(1, 363) = 1.659, *p* = .199; fear of COVID-19 (gender): F(1,363) = 18.819, *p*< .001; fear of COVID-19 (contact x gender): F(1,363) = 5.596, *p* = .019]. The covariate age showed no significant impact (F(1, 363) = 0.364 *p* = .547).]

*c*.*) Financial losses due to COVID-19*. Participants were asked whether they had financial losses in the course of the COVID-19 pandemic. There were 275/368 (74.7%) participants claiming they did not have any financial losses, 93/368 (25.3%) people claiming that they had significant financial losses due to COVID-19.

There is a significant difference between people with no financial losses and people with financial losses concerning their levels of fear of COVID-19. Those who had no financial losses showed lower levels of fear of COVID-19. [ANCOVA: fear of COVID-19 (financial loss): F(2, 363) = 4.948, *p* = .027; fear of COVID-19 (gender):F(1, 363) = 22.853, p< .001; fear of COVID-19 (financial loss x gender): F(1, 363) = 2.953, p< .087. The covariate age showed no significant impact (F(1, 363) = 0.020, *p* = .887.]

*d*.*) Use of social media*. There were 137/386 (37.3%) participants claiming they used not more than 4 different social media platforms and 231/368 (53.7%) participants claiming that they used more than 4 different social media platforms in the last month.

There is no significant difference in participants who use less social media and those who use more social media concerning their levels of fear of COVID-19. [ANCOVA: fear of COVID-19 (social media): F(1, 363) = 0.321, *p* = .571; fear of COVID-19 (gender): F(1, 363) = 20.460, *p*< .001; fear of COVID-19 (social media x gender): F(2, 363) = 0.090, *p* = .764. The covariate age showed no significant impact (F(1, 363) = 0.015 *p* = .904).]

### 4.4. Biopsychosocial model of fear of COVID-19

A multiple regression model was calculated in order to establish a biopsychosocial model of predicting factors of fear of COVID-19. The multiple regression analysis showed an R^2^ of 43.5% and revealed state anxiety (STAI-State), trait anxiety (STAI-Trait), health anxiety (IAS) and female gender as significant predictors of fear of COVID-19 (Severity Measure for Specific Phobia). All other variables (BAI, WI, SPQ, WHO-5, Social Support Survey, Age, Social Media Use, Financial Losses due to COVID-19, social contact with people who were infected with COVID-19, somatic risk factors) were not significant.

Table 5 shows the multiple regression model of fear of COVID-19.

**Table 5:** Multiple regression model of fear of COVID-19.

Modell	Regression coefficient B	Std.-Error	Beta	T	P	VIF
**(Constante)**	-.595	.271		-2.201	.028	1.195
**Gender (Female)**	.165	.064	.119	2.560	**.011**	1.337
**Age**	.001	.003	.024	.487	.627	2.360
**BAI**	.004	.004	.055	.819	.413	2.686
**WHO-5**	.002	.002	.053	.783	.434	3.380
**IAS**	.009	.004	.183	2.402	**.017**	3.380
**STAI-STATE**	.024	.004	.450	5.452	**.000**	3.923
**STAI-TRAIT**	-.010	.005	-.180	-2.040	**.042**	4.342
**WI**	.022	.016	.094	1.356	.176	2.804
**SPQ**	-.295	.162	-.227	-1.815	.070	3.148
**Phobia from medical procedures (SPQ)**	-.062	.138	-.047	-.449	.654	2.641
**Social support**	.001	.002	.041	.797	.426	1.482
**Use of social media**	.056	.058	.044	.963	.336	1.112
**Contact with people who were infected with COVID-19**	-.040	.054	-.033	-.743	.458	1.131
**Somatic risk factors**	.041	.055	.033	.744	.458	1.118
**Financial losses**	.118	.061	.085	1.926	.055	1.112

a. Depending Variable: Severity measure for specific phobia[15] (adapted for fear of COVID-19).

BAI: Beck’s Anxiety Inventory [16].

IAS: Whiteley-Index [18].

Severity Measure for Specific Phobia [15].

Somatic risk factors: obesity, old age, cardiovascular disease, autoimmune disease, cancer, medication that impacts the immune system, liver disease.

SPQ: Specific Phobia Questionnaire [19].

SSS: Social Support Survey [21].

STAI: State-Trait Anxiety Inventory (STAI) [17].

WHO-5: WHO-5 Well-being Index [20].

WI: Whiteley-Index and Illness Attitude Scales [18].

**Fig 2:**
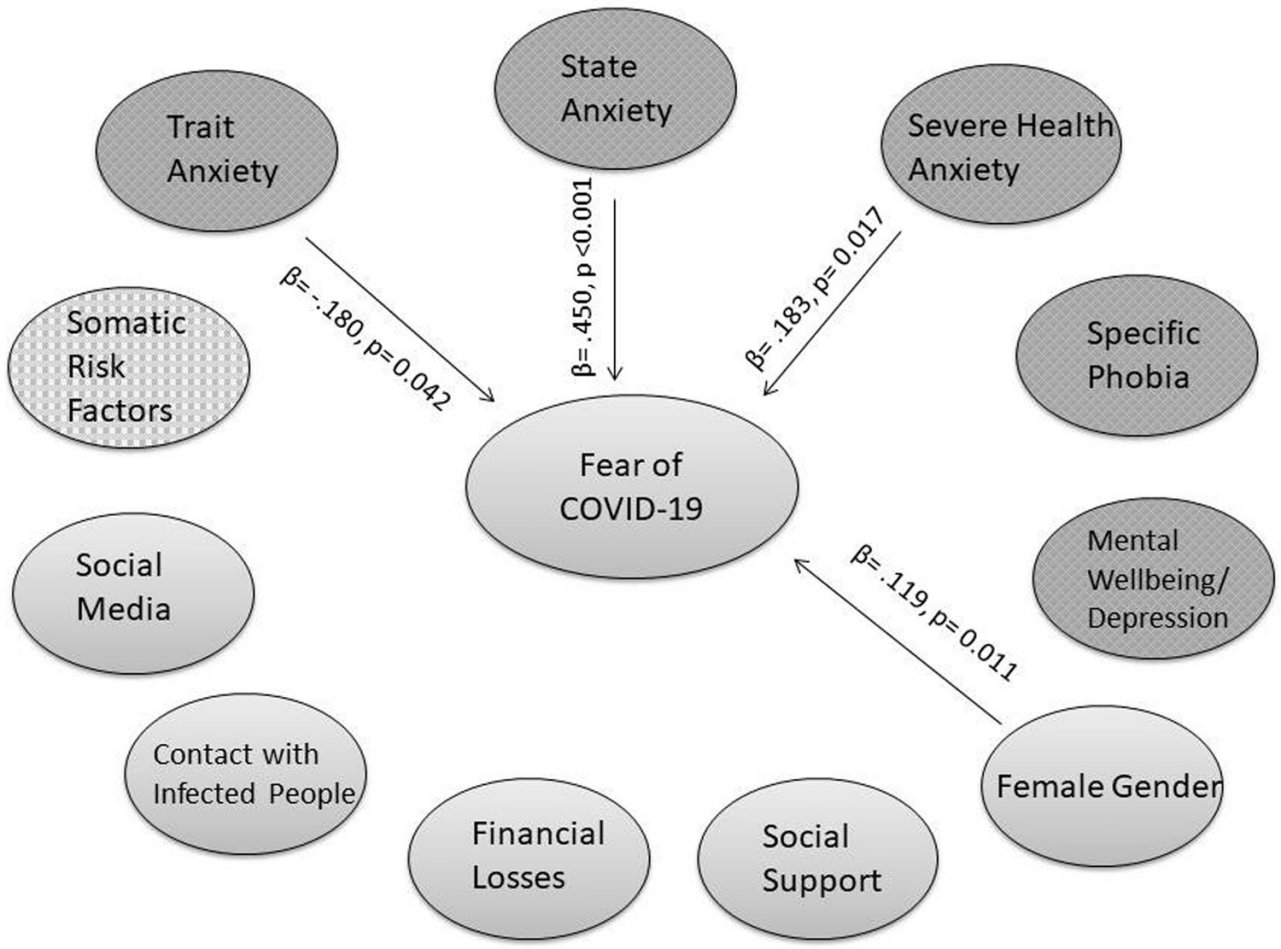
Biopsychosocial model of fear of COVID-19 –Predictors/non predictors in the multiple regression model with B-values and p-values.

**Fig 2** shows a graphic overview of all the predictors (marked with arrows) and non-predictors of fear of COVID-19 and the biopsychosocial model of fear of COVID-19 (Fig 2).

## 5. Discussion

COVID-19 is seen as a direct or indirect threat by many people worldwide and causes a lot of fear. Thus it is important to find biopsychosocial factors that might aggravate fear of COVID-19. Recent studies already show that there is a rise in numbers of patients with psychiatric diseases due to the COVID-19 pandemic, affected are not only health care workers with intensive COVID-19 patients contacts, but also the general public who has been affected by COVID-19 infections, restrictions, lockdowns and the general uncertainty [2, 6, 7, 10]. There are two factors that may influence a person’s mental health negatively. The SARS-CoV2 virus is able to have a direct neurological and psychiatric impact on the infected person, similar to other viruses that may also show such effects [22]. Then, there might be a feeling of uncertainty, maybe combined with misinformation or the feeling of being overwhelmed by negative COVID-19 news that can be consumed on the media, which all may lead to severe fear and uncertainty [23]. Fear is an important mechanism for protection and survival. Evolutionary fear helps animals and humans to react with a fight or flight reaction to real or felt threats to their lives. However, The Yerkes-Dodson law states that there is optimal productivity and effectivity only in a medium range of arousal and activation. This shows that severe fear might have negative effects on a person’s productivity and effectivity [24]. The biopsychosocial model is well established in explaining pathogenesis of psychiatric diseases and thus it is also predestined for characterizing potential psychosocial risk factors that might aggravate fear of COVID-19 [25].

### a.) Biological factors

In this study the pre-existing somatic risk factors did not impact the levels of fear of COVID-19.

### b.) Psychological factors

Considering their psychological risk factors there were significant differences visible between the participants. In this study participants that showed more severe state and trait anxiety (STAI) also showed higher levels of fear of COVID-19. As the COVID-19 pandemic may be seen as a threat by many people, it is understandable that those people who were already in danger of developing fear and other mental health complications before the pandemic are now the ones that are endangered the most. However, even though those people show a higher predefined risk, everyone can be affected by the threats and collateral damages that the COVID-19 pandemic brings [23]. In addition, participants with severe fear of COVID-19 had more physical symptoms of anxiety (BAI) than participants with almost no fear of COVID-19. Similar results were seen in Whitely-Index WI and in the IAS (which both measure severe health anxiety) were participants that showed higher scores in the WI-IAS also showed higher levels of fear of COVID-19. Regarding specific phobias (SPQ) those participants who showed a higher tendency of specific phobias also showed higher levels of fear of COVID-19. Concerning mental wellbeing/signs of depression (WHO-5) those participants who showed higher levels of mental wellbeing and not relevant signs of depression, also showed lower levels of fear of COVID-19. Previous studies showed that depression was often associated with higher levels of fear [26].

### c.) Psychosocial factors

There were no significant differences in the groups who have lower levels of fear of COVID-19 and those who have higher levels of fear of COVID-19 concerning their social support or social media use. The presence of financial losses also made a difference, those people who had to suffer from financial losses due to the COVID-19 pandemic showed higher levels of fear of COVID-19. Financial security seems to be a protective factor for a good health-related quality of life, as financial insecurity or financial losses are associated with higher numbers of mental health problems such as anxiety disorders and depressions [27]. Regarding the impact of previous social contact with people who were infected with COVID-19 on severe fear of COVID-19 this study revealed gender specific results. So for female participants the study results showed that the more contact with people who were infected with COVID-19 they had, the less afraid of the disease they were. The male participants were more afraid of the disease the more contact with people infected with COVID-19 they had. Concerning marital status, people who are in a relationship show significant lower levels of fear than those who are single, widowed or divorced/separated. Previous studies showed that 1.) social support, 2.) to be in a relationship and 3.) to have some close, trusted person around is a protective factor. In contrast being alone and low levels of social support lead to higher levels of fear and other mental health issues [28].

In a multiple regression model to identify significant biopsychosocial predictors, our study results showed some significant predictors that might aggravate fear of COVID-19. Thus, the regression model identified female gender, severe health anxiety (IAS questionnaire), state anxiety (STAI State) as well as trait anxiety (STAI Trait) as significant predictors that might aggravate fear of COVID-19.

It is notable that female gender is a significant predictor that might aggravate fear of COVID-19 as gender differences also play an important role in the somatic course of COVID-19, as people of different genders show different focuses of symptoms and courses of the disease [29, 30]. Female gender is found as a factor for the development of more and more severe anxiety disorders in general [31–33]. This may be the reason why female gender also was found to be significant predictor in this study.

Finding out if there are predicting factors that might aggravate fear of COVID-19, is crucial in order to identify more people that suffer from overwhelming fear at an early stage and to provide better biopsychosocial support as well as preventive measures to improve health related quality of life. Studies have shown that fear of COVID-19 does not only affect mental health, but also somatic health as many people with severe fear of COVID-19 did not go to see a doctor when medically indicated [6–9]. This leads to decreasing numbers of all kinds of somatic diseases during the pandemic, which can only be explained by underdiagnosis and undertreatment. Many people were afraid to get infected with COVID-19 while being inside a hospital or a doctor’s waiting room and thus did not seek medical help even if they would have needed it [8–10].

## 6. Limitations

The study population was limited so it would be beneficial to have further studies with higher numbers of participants. Moreover, all participants were from middle-Europe, mainly Austria and Germany. Studies in other cultural areas may be beneficial to support the theories. Furthermore, our study included a rather young population with a mean age of 33.51 years (SD ±12.17), more data with a study population of higher age might be valuable. The response rates in female participants were higher than in male participants which resulted in a gender distribution that was of higher percentage in female participants. As the study design was planned at an early stage of the COVID-19 pandemic, not much data about somatic biomarkers about the disease was known then. This is why this study primarily focused on the psychological and social factors. However, as more and more biomarkers are being researched, these should also be integrated into future research projects to a greater extent.

## 7. Conclusion

In this study, certain predictors were found to be predictive for developing higher levels of fear of COVID-19, those are: female gender, state anxiety and trait anxiety as well as severe health anxiety. Apart from those predictive factors, there were findings that being in a relationship is a protective factor against severe fear of anxiety. Interestingly, use of social media and social support did not show significant impacts on the levels of fear of COVID-19. The finding of significant predictors of fear of COVID-19 might contribute to detect people who might suffer most from severe, overwhelming fear of COVID-19 at an early stage. According to the Yerkes-Dodson-Law high levels of fear/uncertainty and arousal lead to less productivity, effectivity in combination with impaired quality of life. It is crucial to find out who in the public is more likely to develop overwhelming fear of a disease, to provide better preventive measures and biopsychosocial support for future pandemics which are likely to come.

The study was recorded at clinicaltrials.gov (ID: NCT04359121). Moreover, it was approved by the ethics committee of the Medical University of Graz (ID: 32–354 ex 19/20).

## Supporting information

S1 Checklist (DOCX)

S1 File CoronastudieDatenPlos. Data used in the analysis.
(SAV)

S1 Protocol (DOCX)
