# Supplementary material for: A biopsychosocial model of severe fear of COVID-19
Source: PLoS One. 2022 Feb 28;17(2):e0264357. doi: 10.1371/journal.pone.0264357 (PMC8884481; doi:10.1371/journal.pone.0264357)
Supplement: S1 Protocol — (DOCX) [file pone.0264357.s003.docx]

**Original Research Title: COVID-19 and the development of phobic fears of disease**

**Title of the Paper: A biopsychosocial model of severe fear of COVID-19**

**Introduction:** COVID-19 is a respiratory infection that causes not only somatic health issues, but also frequently psychosocial burdens. The aims of this study were to investigate biopsychosocial factors that might further aggravate fear of COVID-19, and to establish a biopsychosocial model of severe fear of COVID-19.

**Methods:** 368 participants were included in this study. Biopsychosocial factors observed comprised somatic risk factors, mental health factors (fear of Covid-19, state/trait anxiety, physical symptoms of anxiety, severe health anxiety, specific phobias, depression), and psychosocial factors (social support, financial losses, social media consumption, social contacts with COVID-19 infected people). Psychometric questionnaires included State-Trait Anxiety Inventory, Beck’s Anxiety Inventory, Whiteley-Index/Illness Attitude Scales, Secific Phobia Questionnaire, WHO-5 and Social Support Survey.

**Results:** 162/368 (44.0%) participants had almost no fear, 170/368 (46.2%) participants had moderate fear, and 45/368 (12.2%) participants had severe fear of COVID-19. Female participants and participants without relationships showed higher levels of fear of COVID-19 (gender: χ2= 18.47, p<0.001; marital status: χ2 =14.582, p=0.024). Moreover, financial losses due to the COVID-19 were associated with higher levels of fear of COVID-19 [ANCOVA: fear of COVID-19(financial loss x gender): F(1, 363)= 22.853, p< .001]. Furthermore, the level of fear of COVID-19 increased in male participants when they had contact to people who were infected with COVID-19, while in contrast the level of fear of COVID-19 decreased in female participants when they had such contacts [ANCOVA: fear of COVID-19(contact x gender): F(1,363)= 5.596, p= .019]. Multiple regression analysis revealed female gender, severe health anxiety (WI-IAS) and pre-existing state /trait anxiety (STAI) as significant predictors of severe fear of COVID-19.

**Conclusion:** The reasons for severe fear of COVID-19 are multifactorial. In this study significant predictors of severe fear of COVID-19 were female gender, pre-existing state and trait anxiety, as well as severe health anxiety.

Target population: Adults (19-90) who have not yet been infected with COVID-19 (PCR tested positive) in the geographical area of Austria and Germany. All participants received the same battery of questionnaires.

**Aims of the study:** Fear of COVID-19 is a common, reasonable reaction as fear acts as a biological factor against threats. However, this fear may become overwhelming and severely limiting the quality of life of those affected. In order to classify the specific anxiety and phobias against the coronavirus (SARS-CoV-2) and COVID-19, the anxiety against the coronavirus (SARS-CoV-2) and COVID-19, the personal predisposition for anxiety, the general fear of illness, the general fear in other fear-inducing situations and the mood are examined with well-validated psychometric questionnaires. Also surveyed are current and past physical and mental illnesses, the current life situation under corona and COVID-19, and the social support. The psychometric questionnaires applied are used to investigate the development of phobic fears of disease before and after the availability of a vaccination option against COVID-19 in different population groups. After gaining information we will try to develop a biopsychosocial model of fear of COVID-19.

**Study procedure:**

By evaluation different psychological, social and biological factors we will try to find out predictors of severe fear of COVID-19. Better knowledge of the causative factors of phobic fears against infectious diseases can be helpful in the development of suitable preventive strategies and to improve health-related quality of life. In the future this might contribute to new insights for improving the therapeutic strategies. Everyone will be given a battery of author-complied as well as well validated psychiatric questionnaires in order to ask for biological, psychological and social factors that may predict severe fear of COVID-19. The timespan of the study was set to be approximately 1 year. People were recruited by the authors of the study.

**Primary hypothesis:**

H1: COVID-19 triggers phobic anxiety.

Secondary hypotheses:

H2: People with phobic anxieties caused by COVID-19 also have increased other specific phobias.

H3: People with phobic fears caused by COVID-19 have increased anxiety as a relatively persistent personality trait (trait anxiety).

H4: People with phobic fears caused by COVID-19 have increased general fear of illness and hypochondriac anxiety.

H5: People with phobic fears caused by COVID-19 have more panic attack-like symptoms.

H6: People with phobic anxieties caused by COVID-19 have increased depressive symptoms.

H7: People with increased phobic anxiety due to COVID-19 have less social support.

H8: People with previous direct exposure to COVID-19 sufferers have increased phobic anxiety due to COVID-19.

H9: After the availability of a vaccination against COVID-19, there will be a decline in phobic anxiety, panic attacks, general anxiety and depressive symptoms.

**Objectives:** The aim of this study will be to investigate the development of phobic fears of disease before and after the availability of a vaccination option against COVID-19 in different population groups.

Better knowledge of the causative factors of phobic fears of infectious diseases can be used to develop appropriate preventive strategies and to improve the health-related quality of life.

The study was recorded at clinicaltrials.gov (ID: NCT04359121). Moreover, it was approved by the ethics committee of the Medical University of Graz (ID: 32-354 ex 19/20).

368 participants [male n= 93/368 (25.3%), female n= 275/368 (74.7%); mean age: 33.5 years (SD ±12.1)] were included in this study. All participants answered a research battery of questionnaires consisting of author compiled questionnaires for a.) sociodemographic data, b.) use of social media, media consumption, c.) financial losses/job losses due to COVID-19 and d.) somatic risk factors for severe COVID-19 course of disease alongside with well-validated psychometric questionnaires to objectify e.) the amount of fear of COVID-19 (Severity Measure for Specific Phobia(16) adapted for COVID-19), f.) pre-existing anxiety [physical symptoms of anxiety (Beck’s Anxiety Inventory(BAI, 17)), state anxiety and trait anxiety (STAI, State-Trait Anxiety Inventory(18)), severe health anxiety (WI-IAS, Whiteley-Index and Illness Attitude Scales(19))], g.) specific phobias (SPQ, Specific Phobia Questionnaire(20)), h.) mental wellbeing and signs of depression (WHO-5 Well-being Index(21)) and i.) general social support (SSS, Social Support Survey(22)).

Inclusion criteria included people of all genders, aged 19-90, informed consent. Exclusion criteria were: age below 19 or over 90, pre-existing psychiatric disorders, diagnosed cognitive impairment (e.g. dementia), previous positive test for SARS-COV2 (PCR-test). Most of the participants who completed in the study were situated in Austria, but also some participants from Germany were included. The study period was from April 18^th^ 2020 to April 5^th^ 2021. The participants did not receive any financial compensation.

**Milestones:**

…first year Preparation of the Study, collection of data

…second year Collection of data, data analysis

…third year Writing of the doctoral thesis

**Methods:** Prospective study with well-established and validated psychometric questionnaires

**Required:** Basic knowledge in statistics

Available from: Apr 16, 2020

**References:**

Literature Cited

1. Gandhi RT, Lynch JB, Del Rio C. Mild or Moderate Covid-19. New England Journal of Medicine 2020; 383(18):1757–66.

2. Apuke OD, Omar B. Fake news and COVID-19: modelling the predictors of fake news sharing among social media users. Telematics and Informatics 2021; 56:101475. Available from: URL: https://www.ncbi.nlm.nih.gov/pmc/articles/PMC7390799/.

3. Ornell F, Schuch JB, Sordi AO, Kessler FHP. "Pandemic fear" and COVID-19: mental health burden and strategies. Braz J Psychiatry 2020; 42(3):232–5. Available from: URL: https://pubmed.ncbi.nlm.nih.gov/32267343.

4. Metzler B, Siostrzonek P, Binder RK, Bauer A, Reinstadler SJ. Decline of acute coronary syndrome admissions in Austria since the outbreak of COVID-19: the pandemic response causes cardiac collateral damage. Eur Heart J 2020; 41(19):1852–3. Available from: URL: https://pubmed.ncbi.nlm.nih.gov/32297932.

5. Bugger H, Gollmer J, Pregartner G, Wünsch G, Berghold A, Zirlik A et al. Complications and mortality of cardiovascular emergency admissions during COVID-19 associated restrictive measures. PLoS One 2020; 15(9):e0239801-e0239801. Available from: URL: https://pubmed.ncbi.nlm.nih.gov/32970774.

6. Saraswathi I, Saikarthik J, Senthil Kumar K, Madhan Srinivasan K, Ardhanaari M, Gunapriya R. Impact of COVID-19 outbreak on the mental health status of undergraduate medical students in a COVID-19 treating medical college: a prospective longitudinal study. PeerJ 2020; 8:e10164-e10164. Available from: URL: https://pubmed.ncbi.nlm.nih.gov/33088628.

7. Bueno-Notivol J, Gracia-García P, Olaya B, Lasheras I, López-Antón R, Santabárbara J. Prevalence of depression during the COVID-19 outbreak: A meta-analysis of community-based studies. International Journal of Clinical and Health Psychology 2021; 21(1):100196. Available from: URL: http://www.sciencedirect.com/science/article/pii/S1697260020300545.

8. Dong L, Bouey J. Public Mental Health Crisis during COVID-19 Pandemic, China. Emerg Infect Dis 2020; 26(7):1616–8. Available from: URL: https://pubmed.ncbi.nlm.nih.gov/32202993.

9. Vanhaecht K, Seys D, Bruyneel L, Cox B, Kaesemans G, Cloet M et al. COVID-19 is having a destructive impact on health-care workers' mental well-being. Int J Qual Health Care 2021; 33(1).

10. Du J, Mayer G, Hummel S, Oetjen N, Gronewold N, Zafar A et al. Mental Health Burden in Different Professions During the Final Stage of the COVID-19 Lockdown in China: Cross-sectional Survey Study. J Med Internet Res 2020; 22(12):e24240.

11. Hossain MM, Tasnim S, Sultana A, Faizah F, Mazumder H, Zou L et al. Epidemiology of mental health problems in COVID-19: a review. F1000Res 2020; 9:636. Available from: URL: https://pubmed.ncbi.nlm.nih.gov/33093946.

12. Sampaio F, Sequeira C, Teixeira L. Impact of COVID-19 outbreak on nurses' mental health: A prospective cohort study. Environ Res 2021; 194:110620. Available from: URL: https://pubmed.ncbi.nlm.nih.gov/33316228.

13. Alnazly E, Khraisat OM, Al-Bashaireh AM, Bryant CL. Anxiety, depression, stress, fear and social support during COVID-19 pandemic among Jordanian healthcare workers. PLoS One 2021; 16(3):e0247679-e0247679. Available from: URL: https://pubmed.ncbi.nlm.nih.gov/33711026.

14. Li X, Liu Q. Social Media Use, eHealth Literacy, Disease Knowledge, and Preventive Behaviors in the COVID-19 Pandemic: Cross-Sectional Study on Chinese Netizens. J Med Internet Res 2020; 22(10):e19684-e19684. Available from: URL: https://pubmed.ncbi.nlm.nih.gov/33006940.

15. González-Padilla DA, Tortolero-Blanco L. Social media influence in the COVID-19 Pandemic. Int Braz J Urol 2020; 46(suppl.1):120–4. Available from: URL: https://pubmed.ncbi.nlm.nih.gov/32550706.

16. Craske M et al. Severity Measure for Specific Phobia; 2013.

17. Steer, R. A., & Beck, A. T. Beck Anxiety Inventory: In C. P. Zalaquett & R. J. Wood (Eds.): Scarecrow Education; 1997 1997.

18. Spielberger C.D., Gorsuch, R.L. Manual for the State-Trait Anxiety Inventory. USA: Consulting Psychologists Press; 1983.

19. Hiller W RW. Internationale Skalen für Hypochondire, Deutschsprachige Adaptation des Whiteley-Index (WI) und der Attitude Scales (IAS): Bern; 2004 2004.

20. Ovanessian, M.M., Fairbrother, N.m Vorstenbosch, V. et al. Psychometric Properties and Clinical Utility of the Specific Phobia Questionnaire in an Anxiety Disorders Sample.; 2019 2019.

21. Topp C.W., Østergaard S.D., Søndergaard S., & Bech P. The WHO-5 Well-Being Index: A Systematic Review of the Literature.; 2015 2015.

22. Sherbourne, C.D., Stewart, A.L. The MOS social support survey.; 1991.

23. Papadimitriou G. The "Biopsychosocial Model": 40 years of application in Psychiatry. Psychiatriki 2017; 28(2):107–10.

24. Mukherjee S, Pahan K. Is COVID-19 Gender-sensitive? J Neuroimmune Pharmacol 2021; 16(1):38–47. Available from: URL: https://pubmed.ncbi.nlm.nih.gov/33405098.

25. Liu N, Zhang F, Wei C, Jia Y, Shang Z, Sun L et al. Prevalence and predictors of PTSS during COVID-19 outbreak in China hardest-hit areas: Gender differences matter. Psychiatry Res 2020; 287:112921. Available from: URL: https://pubmed.ncbi.nlm.nih.gov/32240896.
